# Genetic Profiling and Survival Outcomes in Romanian Colorectal Cancer Patients

**DOI:** 10.7759/cureus.62390

**Published:** 2024-06-14

**Authors:** Alexandra Vesa, Octavian Maghiar, Ovidiu Pop, Monica Boros, Andrei Pascalau, Otto Molnar, Adrian Maghiar

**Affiliations:** 1 Morphological Sciences, University of Oradea, Faculty of Medicine and Pharmacy, Oradea, ROU; 2 Surgical Sciences, University of Oradea, Faculty of Medicine and Pharmacy, Oradea, ROU; 3 Doctoral Studies (Biomedical Sciences), University of Oradea, Faculty of Medicine and Pharmacy, Oradea, ROU; 4 Surgery Sciences, University of Oradea, Faculty of Medicine and Pharmacy, Oradea, ROU

**Keywords:** braf mutation, kras mutation, genetic profiling, mortality, overall survival (os), colorectal cancer

## Abstract

Background: The increasing incidence of colorectal cancer is one of the most frequently addressed medical topics worldwide. It represents the third most commonly diagnosed cancer in both men and women globally, with significant implications for public health. Mortality for this type of malignancy remains high, second only to lung cancer. Given their clinical relevance, the identification and understanding of KRAS and BRAF mutations have become crucial components of personalized medicine approaches in colorectal cancer. Hence, our desire is to carry out a research that analyzes the impact of these mutations in terms of survival and mortality on patients diagnosed with colorectal cancer.

Methods: We conducted a retrospective study spanning from 2018 to 2022, which involved 118 patients diagnosed with colorectal cancer. The patients were selected from the databases of the Oradea County Emergency Clinical Hospital and Pelican Oradea Hospital. Genetic testing was performed at the "Resident Laboratory" clinic. Subsequently, patients were divided into two groups of equal size based on the presence or absence of mutations.

Results: The survival rate one year after the diagnosis of colorectal cancer is 84.74% (N=50/59) for the mutant group versus 67.96% (N=40/59) for the wild-type group. The survival rate at five years from the diagnosis of colorectal cancer is 25.93% (N=15/59) for patients with wild-type tumors compared to 33.54% (N=20/59) for patients with tumors with mutant status (p=0.483, HR=1.153, CI 95% 0.7661-1.735). The five-year survival rate, depending on the mutation present, highlights the fact that the average overall survival for those with the KRAS mutation is 38.6 months (CI 95% 35.22-41.97) and for those with the BRAF mutation is 8.3 months (CI 95% 5.42-11.17) (p=0.039). The mortality rate for mutant KRAS is 44.89% (N=22/50), while for those with mutant BRAF, it is 100% (N=6/6).

Conclusions: We observed no statistically significant difference in overall survival rate and disease-free interval between the two studied groups, but the overall survival was better for those with mutations present (38.64 months versus 31.07 months for wild-type tumors). The mortality rate is higher among tumors with wild-type status (p=0.005), in the first year after the diagnosis of colorectal cancer. The BRAF mutation confers a much worse prognosis than the KRAS mutation, from both the survival analysis and the mortality rate.

## Introduction

Colorectal cancer (CRC) represents a significant public health challenge globally, with increasing incidence rates observed in recent years. In Romania, as in many other countries, CRC remains a leading cause of cancer-related morbidity and mortality. Understanding the molecular mechanisms underlying CRC progression and their impact on patient outcomes is crucial for the development of effective personalized treatment strategies. Genetic profiling has emerged as a powerful tool in this regard, allowing for the identification of key genetic alterations associated with CRC pathogenesis and prognosis.

The complex nature of the disease results from a multitude of factors, with genetic mutations playing an essential role in its pathogenesis. Our genetic makeup, inherited from our parents, contains instructions that govern various cellular processes, including cell growth, division, and repair. When mutations occur in certain genes responsible for regulating these processes, the balance is disrupted, leading to an uncontrolled proliferation of cancer cells. Among the many genetic alterations involved in CRC, mutations in the KRAS and BRAF genes have emerged as crucial factors in tumor development and progression.

According to GLOBOCAN 2022 data, CRC in Romania ranks first in incidence regardless of gender, with a total of 13,541 newly diagnosed cases. It occupies the third position in frequency among males after prostate and bronchopulmonary cancer (8,056 newly diagnosed cases) and the second position in frequency among females after breast cancer (5,485 newly diagnosed cases). In terms of mortality rate, CRC ranks second, with a total of 7,381 cases, highlighting that over half of the newly diagnosed cases are followed by death [1].

CRC was among the earliest tumors to undergo comprehensive investigation into the diverse genes and pathways implicated in its onset and advancement [2]. RAS mutation is the most common oncogenic alteration in human cancers. KRAS is the most frequently mutated, followed by NRAS. Flagship KRAS mutant cancers are pancreatic, colorectal, lung, and urogenital adenocarcinomas [3]. It is known that approximately 30-50% of colorectal tumors have a mutated KRAS gene and approximately 5-10% of cases have a mutated BRAF gene [4].

The aim of this study is to comprehensively analyze the genetic profiles of CRC patients and correlate them with survival outcomes, with the specific objectives of identifying genetic mutations associated with prognosis, assessing their impact on patient survival, and elucidating potential biomarkers for risk stratification and personalized treatment approaches. Additionally, the study seeks to evaluate the influence of demographic and clinical factors on survival outcomes in this population, providing valuable insights into the genetic determinants of CRC progression and prognosis in the Romanian context.

## Materials and methods

We conducted a retrospective study in the period 2018-2022, including 118 patients diagnosed with CRC. The patients were selected from the database of the Oradea County Emergency Clinical Hospital, of the Municipal Clinical Hospital "Dr. Gavril Curteanu" Oradea, and Pelican Oradea Hospital, with the agreement of the management of the mentioned institutions. The genetic tests were carried out at the "Resident Laboratory" clinic, also with the consent of the management to access the database.

The patients were subsequently divided into two groups equal in number of patients, depending on the presence or absence of mutations, namely, the group without mutations present called "Group A wild-type" and the group with mutations called "Group B mutant," respectively.

Inclusion criteria were as follows: age over 18 years, histopathological diagnosis of CRC, patients with genetic testing performed from 2018 to 2022 inclusive, tumors with microsatellite stability (MS-S), and consent of the patient or relatives (in case of death) to participate in the study. In contrast, exclusion criteria were the following: colorectal tumor diagnosis based only on imaging investigations, without a definite histopathological result, genetic testing with inconclusive result, non-compliant patients or patients' refusal to participate in the study, and histopathological diagnosis different from adenocarcinoma.

The genetic testing was carried out through an automatic real-time PCR method, according to the "cascade" algorithm, which includes three stages: Stage 1 is the KRAS exon 2 screening which involves testing for seven mutations located at codons 12 and 13. If a mutation is identified, further testing is discontinued, rendering the patient ineligible for personalized therapy. However, if no mutation is detected, the testing proceeds to step 2. Stage 2 is the extended RAS testing which includes the examination of 28 mutations within exons 3 and 4 of the KRAS gene, as well as exons 2, 3, and 4 of the NRAS gene. Stage 3 is the BRAF mutation testing which enables the identification of five mutations occurring at codon 600, including the V600E mutation.

Mutational analysis typically involves processing formaldehyde-fixed paraffin-embedded tissues, wherein paraffin is removed and DNA extraction is carried out using standardized protocols. In the testing of KRAS mutations, PCR amplification techniques are used in the first stage. Depending on the analyzed tissue, the ratio of tumor tissue to healthy tissue is variable and heterogeneous, resulting in a mixture of the target for amplification in which mutant DNA and wild-type DNA are not present in an equimolar ratio. Therefore, it is important that the selected tissue for genotyping contains sufficient tumor material for analysis (more than 70% invasive carcinoma cells).

Disease-free interval (DFI) was defined as the interval between CRC diagnosis or registration in the study and disease progression (occurrence of local recurrences and/or metastases). Survival rate (SR) was defined as the interval between diagnosis or registration in the study and death from any cause. The mortality rate is defined as a measure of the frequency of death in cohort patients within the study-specific interval.

The overall survival (OS) and DFI were estimated using the Kaplan-Meier method, and the comparison of the two groups was applied by the log-rank test, with the use of GraphPad Prism version 10.0.0 for Windows (GraphPad Software, Boston, Massachusetts USA, www.graphpad.com). Besides the Kaplan-Meier analysis, a t-test was used to determine if there is a significant difference between the means of the two groups and how they are related.

## Results

In our study, we observed that the predominant mutation is the KRAS mutation (83.05%, N=49/59). Additionally, NRAS (3.38%, N=2/59), BRAF (10.16%, N=6/59), and concurrent KRAS/BRAF mutations (3.38%, N=2/59) were also detected. The ANOVA test conducted revealed a significant difference in the standard deviation (p<0.05).

**Figure 1:**
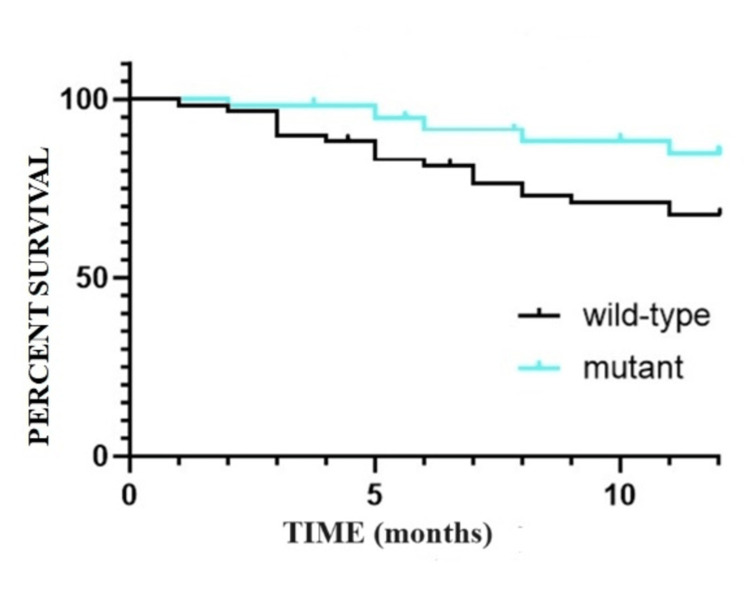
Overall survival at one year: Kaplan-Meier curve.

**Figure 2:**
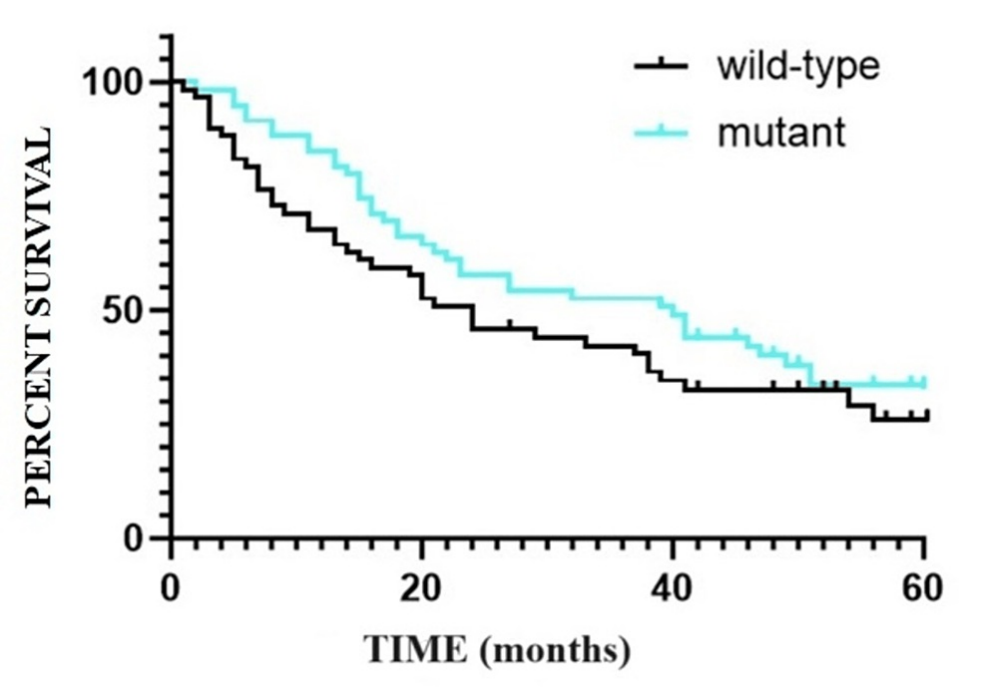
Overall survival at five years: Kaplan-Meier curve.

The findings regarding OS are depicted in Figure 1 and Figure 2. Notably, one year after the diagnosis of CRC, the OS is 84.74% (N=50/59) for the mutant group, contrasting with 67.96% (N=40/59) for the wild-type group. Similarly, the five-year SR following CRC diagnosis is 25.93% (N=15/59) for patients with wild-type tumors, compared to 33.54% (N=20/59) for patients with tumors exhibiting mutant status. In terms of comparing survival curves, the obtained values include p=0.483, HR=1.153, and CI 95% ranging from 0.7661 to 1.735 (based on the log-rank test).

Furthermore, the median OS rate for Group A, characterized by the wild-type genotype, is 31.07 months (with a CI 95% of 27.291-38.489), while for Group B, exhibiting the mutant genotype, it is 38.64 months (with a CI 95% of 35.061-42.219). 

The OS diminishes with the passage of time following the initial diagnosis of CRC. While the SR at one year exhibits superior values for the mutant lot, a trend towards convergence in SRs for both groups becomes apparent from the second year onwards (Table 1).

**Table 1:** OS 1-5 years. OS: overall survival; N: number; CI: confidence interval; *: p-value; t-test: p=0.270; CI 95%: -15.49 to 37.18

OS	Group A wild-type		Group B mutant	
	%	N	%	N
0-12 months	67.96%	40	84.74%	50
12-24 months	45.76%	27	57.62%	34
24-36 months	42.16%	25	52.54%	31
36-48 months	32.41%	19	40.02%	24
48-60 months	25.93%	15	33.54%	20

**Figure 3:**
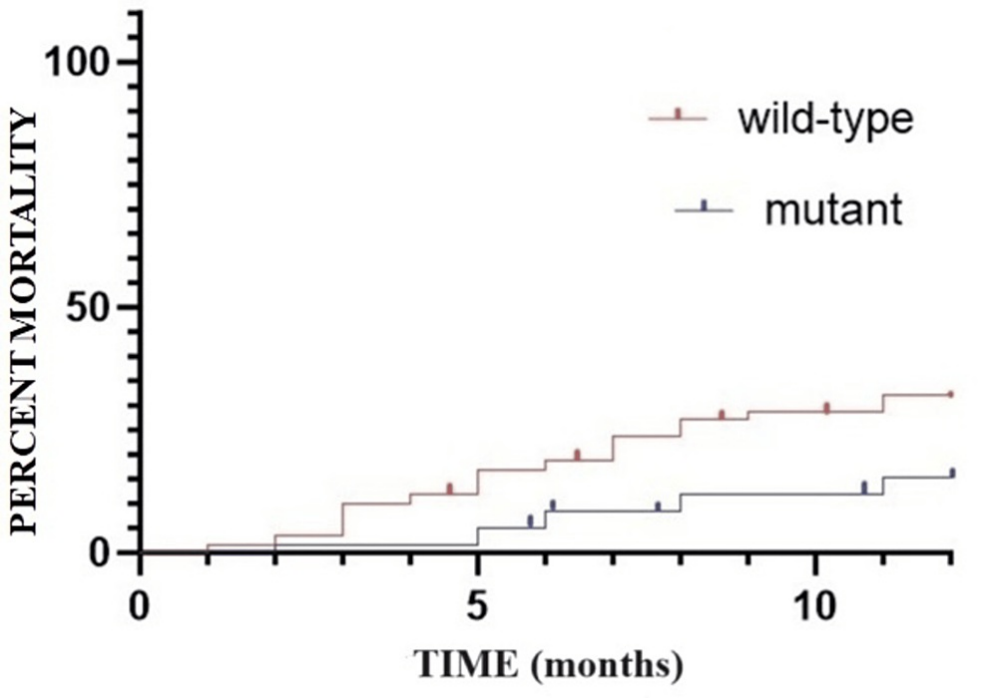
Mortality rate in the first year after the initial diagnosis of colorectal cancer: Kaplan-Meier curve.

**Figure 4:**
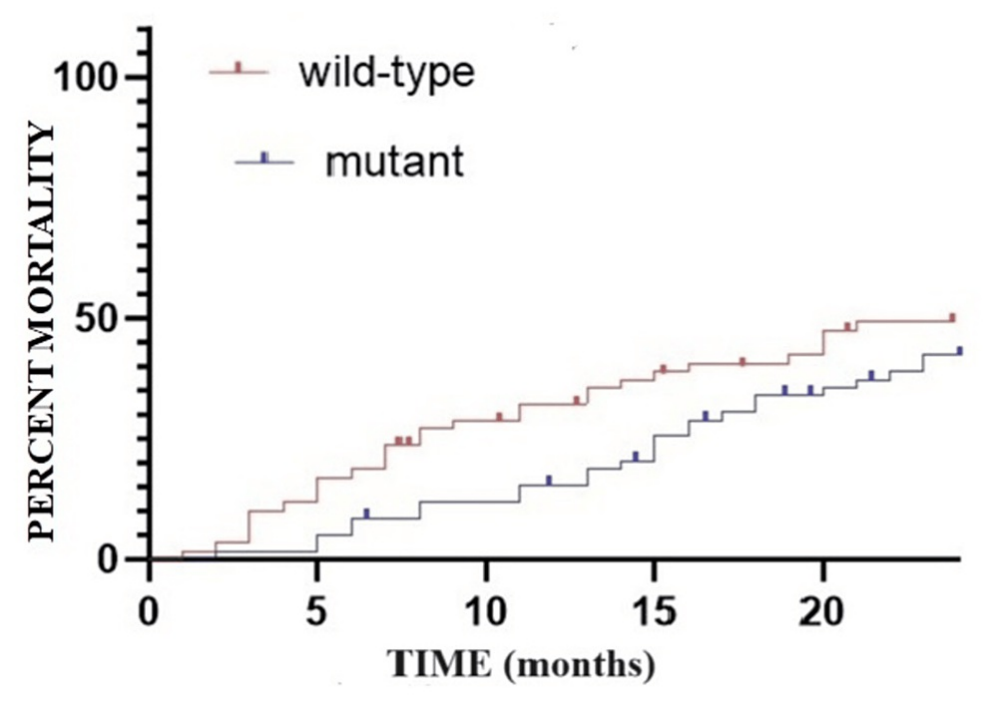
Mortality rate at two years after the initial diagnosis of colorectal cancer: Kaplan-Meier curve.

The probability of death within the initial 12 months post-diagnosis is higher in the wild-type group compared to the mutant group: 32.30% (N=19/59) in the wild-type group versus 15.25% (N=9/59) in the mutant group. By the 24-month mark, there is an observable trend towards equalizing mortality rates between the two groups; however, mortality remains notably higher in the wild-type group: 54.23% (N=32/59) in the wild-type group versus 42.37% (N=25/59) in the mutant group. The t-test conducted to calculate the probability of death between the two groups over a five-year period yields a p-value of 0.054, indicating statistically significant differences between the two groups (Figure 3, Figure 4, and Table 2).

**Table 2:** The mortality rate in the first five years since the initial diagnosis according to the group of patients. N: number; *: p-value; t-test: p=0.0054; CI 95%: -15.85 to -5.194

Elapsed time since the initial diagnosis	Group A wild-type		Group B mutant	
	%	N	%	N
0-12 months	32.30%	19	15.25%	9
12-24 months	54.23%	32	42.37%	25
24-36 months	57.83%	34	47.45%	28
36-48 months	67.58%	40	59.97%	35
48-60 months	74.06%	44	69.24%	41

**Figure 5:**
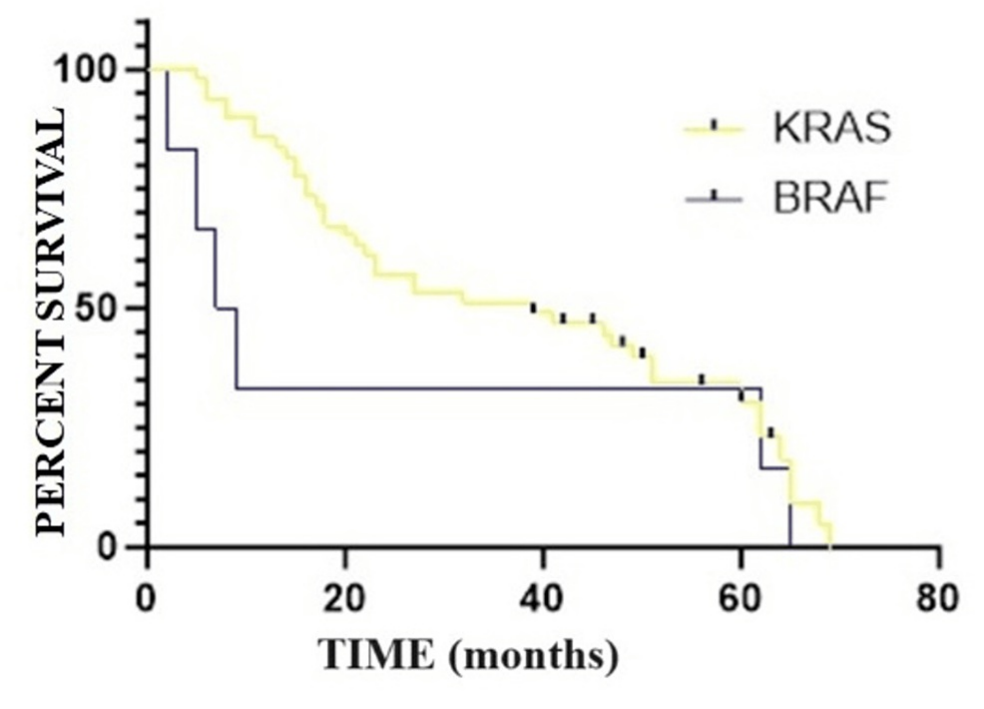
Survival analysis based on the type of mutation present: Kaplan-Meier curve.

The five-year SR, depending on the mutation present, highlights the fact that the average OS for those with the KRAS mutation is 38.6 months (CI 95% 35.22-41.97) and for those with the BRAF mutation is 8.3 months (CI 95% 5.42-11.17). The Gehan-Breslow-Wilcoxon analysis shows a p-value of 0.039, which denotes a significant difference in terms of survival for mutant KRAS patients and survival for mutant BRAF patients (Figure 5). The mortality rate for mutant KRAS is 44.89% (N=22/50), while for those with mutant BRAF, it is 100% (N=6/6).

**Figure 6:**
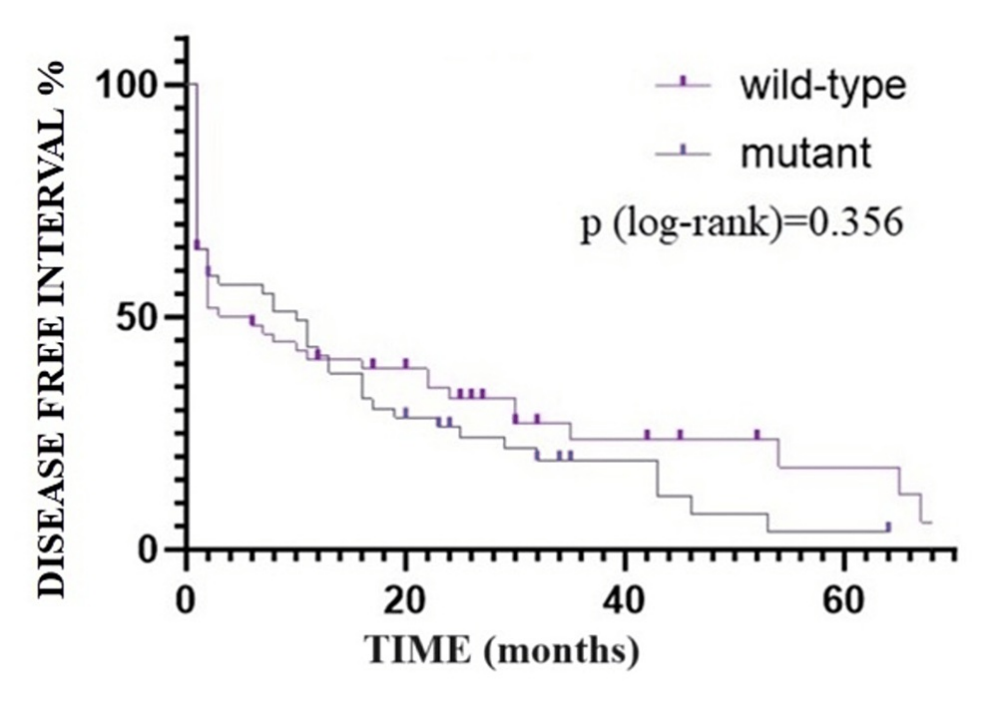
Survival analysis of disease-free interval: Kaplan-Meier curve.

In the initial 12 months following the primary diagnosis, patients with mutant tumors exhibit a prolonged DFI compared to those in the opposing group: 57.11% (N=34/59) for mutant tumors versus 50.16% (N=30/59) for wild-type tumors. Subsequently, beyond this timeframe (>12 months), we observe a sharp decline in the DFI among patients with mutant tumors in contrast to those with wild-type tumors, albeit without statistically significant differences between the two groups (p-value=0.345, according to the log-rank test) (Figure 6). Despite this notable disparity between the two groups, at the end of the study period following the initial diagnosis, no patients (N=0/59) from the wild-type group experienced DFI in contrast to 3.85% (N=2/59) of patients with mutant tumors.

The median DFI for Group A, comprising wild-type tumors, is six months (CI 95% 3.357-8.463), while for Group B, consisting of mutant tumors, it is 10 months (CI 95% 7.737-12.263).

## Discussion

While KRAS mutations have been identified as crucial predictive factors, their prognostic significance continues to undergo evaluation. Numerous studies have underscored the unfavorable prognosis of CRC patients harboring KRAS mutations [5]. For instance, a study conducted by Wang et al. on a cohort of 1655 patients found that KRAS mutant CRC is linked with an unfavorable prognosis (HR=1.30, p=0.008), necessitating the utilization of distinct prognostic markers [6]. Studies involving both KRAS mutation and BRAF mutation were carried out by Modest et al., de Cuba et al., and Wang et al. (the median OS for patients with WT, KRAS, NRAS, and BRAF was 49.2, 36.2, 30.1, and 22.5 months) [7-9].

According to Alkader and colleagues, the median OS for patients with KRAS mutation was 17 months (95% CI: 13.762-19.273), whereas for patients with wild-type KRAS, it was 21 months (95% CI: 20.507-27.648) [10]. This study approaches the number of investigated patients with those included in our study (N=135). There is a difference with the results obtained by us, the median global SR for KRAS wild-type being 28 months (95% CI 0.4031-0.9396) and for mutant KRAS being 39 months (95% CI 1.064-2.481), but without a significant difference (p log-rank=0.630, HR=1.106, CI 95% 0.7249-1.688). The result obtained by us is corroborated with the result obtained by Won et al. who concluded that no significant difference was revealed in SR and DFI depending on the KRAS mutation status (p=0.108) [11].

Another study with approximately the same number of patients as those in our study (N=113) and with a similar result is the one conducted by Promsorn and colleagues, where it was demonstrated that there were no significant differences in OS between patients with KRAS mutations and those without (p=0.159) but with the mention that those in the KRAS mutation group had a lower SR [12]. A similar result was obtained by the research of Habashy et al., in which OS did not differ significantly between patients with wild-type and mutant KRAS [13]. Also, Baek et al. in a recent 2022 study found that KRAS mutation status was not correlated with survival [14].

Overall, the true role of KRAS mutation status in CRC survival is still debated, and most of the scientific literature seems against its prognostic role [15].

Regarding the SR for the BRAF mutation, our results are similar to those in the specialized literature [7,9,11] in the sense that its presence confers a much worse prognosis compared with the KRAS mutation (p=0.039). The adverse impact of BRAF mutation was reported in the AIO study KRK0207, in which BRAF mutation was reported to be the strongest adverse prognostic factor (HR 3.16; 95% CI 2.17-4.60; p<0.0001) compared to RAS status and primary tumor location [16].

In our study, we observed that within the first 12 months following the initial diagnosis of CRC, both the SR and mortality were notably better for patients with mutant tumors compared to those with wild-type tumors. This finding contrasts with previous studies, which reported a generally poorer prognosis for patients with KRAS mutations. One possible explanation for this discrepancy could be related to differences in patient demographics, treatment protocols, or even regional variations in healthcare practices.

What if the early survival advantage observed in our mutant group is attributable to specific, yet unidentified, genetic or environmental factors unique to our study population? Additionally, it is worth considering that early interventions or aggressive treatments administered more promptly to patients with mutant tumors might have contributed to their initial survival benefit. What if the improved early survival in the mutant group indicates a transient benefit from certain chemotherapeutic regimens that these patients received, which might be more effective during the initial stages of the disease?

This early advantage could also suggest that the biological behavior of mutant tumors, particularly in the short term, might be more amenable to current therapeutic approaches. Another possibility is that the healthcare providers managing our cohort had a higher index of suspicion and, consequently, initiated treatment more rapidly for mutant cases due to an awareness of their typically poorer long-term prognosis, inadvertently providing an early survival boost.

Moreover, what if this initial survival benefit diminishes over time due to the inherent aggressive nature of mutant tumors, which might lead to more rapid disease progression post the first year? This hypothesis is supported by our observation that the survival curves tend to equalize around the 24-month mark, indicating a potential shift in the disease dynamics as the duration from diagnosis increases.

Further investigation is needed to validate these hypotheses and understand the underlying mechanisms driving these differences. Our findings underscore the complexity of CRC prognosis and highlight the importance of individualized patient management based on genetic profiling. Future studies should aim to dissect these early survival benefits and explore whether they can be sustained or even enhanced through tailored therapeutic approaches. 

Limitations of the study

The study comprises 118 patients, which may not be representative of the larger population of CRC patients in Romania. Also, the study may not account for all potential confounding factors, such as comorbidities, lifestyle factors, and socioeconomic status.

## Conclusions

There is no statistically significant difference in OS rate and DFI between the two studied groups. Tumors with wild-type status exhibited a higher mortality rate (p=0.005), with the mortality rate in this group being twice that of the opposite group within the first year following the diagnosis of CRC. Similar to the analysis of the SRs, at the 24-month mark, we notice a trend towards equalizing mortality rates between the two groups, although mortality remains elevated in the wild-type group (54.23% (N=32/59) in the wild-type group versus 42.37% (N=32/59) in the mutant group). The BRAF mutation confers a much worse prognosis than the KRAS mutation, from both the SR analysis and mortality rate (which in our study was 100% (N=6/6)).

Based on our study, it seems that patients with mutant tumors may have better outcomes in the first 12 months after being diagnosed with CRC compared to those with wild-type tumors. This suggests that the mutational status could be a useful indicator for short-term prognosis, affecting treatment decisions. However, further research is needed to confirm these findings and understand why there are differences in outcomes between mutant and wild-type tumors. Clinicians may want to monitor and provide personalized interventions for patients with wild-type tumors during the critical first 12 months after diagnosis to improve SRs.

## References

[1] Ferlay J, Ervik M, Lam F (2024). Data visualization tools for exploring the global cancer burden in 2022. https://gco.iarc.who.int/today/en.

[2] Ozdemir Y, Cag M, Colak E (2021). The effect of gene mutations on metastasis and overall survival in metastatic and nonmetastatic colon cancers. Asian Pac J Cancer Prev.

[3] Timar J, Kashofer K (2020). Molecular epidemiology and diagnostics of KRAS mutations in human cancer. Cancer Metastasis Rev.

[4] Maurie Markman (2022). Colorectal cancer and KRAS/BRAF. January.

[5] Koulouridi A, Karagianni M, Messaritakis I (2022). Prognostic value of KRAS mutations in colorectal cancer patients. Cancers (Basel).

[6] Wang J, Song J, Liu Z, Zhang T, Liu Y (2022). High tumor mutation burden indicates better prognosis in colorectal cancer patients with KRAS mutations. Front Oncol.

[7] Modest DP, Ricard I, Heinemann V (2016). Outcome according to KRAS-, NRAS- and BRAF-mutation as well as KRAS mutation variants: pooled analysis of five randomized trials in metastatic colorectal cancer by the AIO colorectal cancer study group. Ann Oncol.

[8] de Cuba EM, Snaebjornsson P, Heideman DA (2016). Prognostic value of BRAF and KRAS mutation status in stage II and III microsatellite instable colon cancers. Int J Cancer.

[9] Wang Y, Loree JM, Yu C (2018). Distinct impacts of KRAS, NRAS and BRAF mutations on survival of patients with metastatic colorectal cancer. J Clin Oncol.

[10] Alkader MS, Altaha RZ, Badwan SA (2023). Impact of KRAS mutation on survival outcome of patients with metastatic colorectal cancer in Jordan. Cureus.

[11] Won DD, Lee JI, Lee IK, Oh ST, Jung ES, Lee SH (2017). The prognostic significance of KRAS and BRAF mutation status in Korean colorectal cancer patients. BMC Cancer.

[12] Promsorn J, Chadbunchachai P, Somsap K (2021). Imaging features associated with survival outcomes among colorectal cancer patients with and without KRAS mutation. Egypt J Radiol Nucl Med.

[13] Habashy P, Lea V, Wilkinson K (2023). KRAS and BRAF mutation rates and survival outcomes in colorectal cancer in an ethnically diverse patient cohort. Int J Mol Sci.

[14] Baek JH, Kim J, Baek DW (2022). Clinical implication of KRAS mutation variants in patients with resected colon cancer. Cancer Diagn Progn.

[15] Cefalì M, Epistolio S, Palmarocchi MC, Frattini M, De Dosso S (2021). Research progress on KRAS mutations in colorectal cancer. J Cancer Metastasis Treat.

[16] Hegewisch-Becker S, Nöpel-Dünnebacke S, Hinke A (2018). Impact of primary tumour location and RAS/BRAF mutational status in metastatic colorectal cancer treated with first-line regimens containing oxaliplatin and bevacizumab: prognostic factors from the AIO KRK0207 first-line and maintenance therapy trial. Eur J Cancer.

